# Differential Estrogen-Regulation of CXCL12 Chemokine Receptors, CXCR4 and CXCR7, Contributes to the Growth Effect of Estrogens in Breast Cancer Cells

**DOI:** 10.1371/journal.pone.0020898

**Published:** 2011-06-10

**Authors:** Antoine Boudot, Gwenneg Kerdivel, Denis Habauzit, Jerome Eeckhoute, François Le Dily, Gilles Flouriot, Michel Samson, Farzad Pakdel

**Affiliations:** 1 UMR CNRS 6026, Molecular and Cellular Interactions, IRSET, University of Rennes 1, IFR 140, Rennes, France; 2 EA4427 SeRAIC, IRSET, University of Rennes 1, IFR 140, Rennes, France; Institut de Génomique Fonctionnelle de Lyon, France

## Abstract

CXCR4 and CXCR7 are the two receptors for the chemokine CXCL12, a key mediator of the growth effect of estrogens (E2) in estrogen receptor (ER)-positive breast cancers. In this study we examined E2-regulation of the CXCL12 axis components and their involvement in the growth of breast cancer cells. CXCR4 and CXCR7 were differentially regulated by E2 which enhanced the expression of both CXCL12 and CXCR4 but repressed the expression of CXCR7. Formaldehyde-associated isolation of regulatory elements (FAIRE) revealed that E2-mediated transcriptional regulation of these genes is linked to the control of the compaction state of chromatin at their promoters. This effect could be accomplished *via* several distal ER-binding sites in the regions surrounding these genes, all of which are located 20–250 kb from the transcription start site. Furthermore, individual down-regulation of CXCL12, CXCR4 or CXCR7 expression as well as the inhibition of their activity significantly decreases the rate of basal cell growth. In contrast, E2-induced cell growth was differentially affected. Unlike CXCR7, the inhibition of the expression or activity of either CXCL12 or CXCR4 significantly blunted the E2-mediated stimulation of cellular growth. Besides, CXCR7 over-expression increased the basal MCF-7 cell growth rate and decreased the growth effect of E2. These findings indicate that E2 regulation of the CXCL12 signaling axis is important for the E2-mediated growth effect of breast cancer cells. These data also provide support for distinct biological functions of CXCR4 and CXCR7 and suggest that targeting CXCR4 and/or CXCR7 would have distinct molecular effects on ER-positive breast tumors.

## Introduction

Estrogens, notably 17-β-estradiol (E2), play a crucial role in the control of epithelial cell proliferation and the differentiation of normal mammary gland cells as well as in breast carcinomas. The effects of E2 are mediated principally by nuclear estrogen receptors alpha (ERα) and beta (ERβ). ERα is expressed in 70% of diagnosed breast cancers [1], [2]. In ER-positive breast cancer cells, E2 stimulates cell growth and plays a role in cancer progression [3]. The proliferative effect of E2 can be repressed using anti-estrogens used clinically in hormono-therapy [4]. Moreover, ER-positive breast tumors appear to be more differentiated and appear to metastasize less than ER-negative breast tumors [5], [6]. Thus, the expression of ERα in breast tumors is generally considered to be an indicator for a good prognosis.

Although recent works reported that the expression of many genes are regulated by E2 in ER-positive breast cancer cells, little is known about their role in E2-growth effect [3], [7]. The chemokine CXCL12 (also named SDF-1 for Stromal-cell Derived Factor 1) was identified as a key mediator of E2-induced breast cancer cell proliferation and survival [8], [9]. This chemokine has several well-known functions: (i) in cell migration during embryonic development, (ii) in the chemotactism of circulating leucocytes and (iii) in the homing of hematopoietic stem cells in bone marrow niches [10], [11]. Moreover, CXCL12 regulates the homeostasis, angiogenesis, proliferation, survival and migration of cancer cells [12], [13], [14]. The G Protein-Coupled-Receptor (GPCR) CXCR4, an E2 endogenous target in endometrial cancer cells [15], binds CXCL12. The high expression of CXCR4 has been often associated with an invasive and migratory phenotype of cancer cells [16], [17]. Metastatic breast tumor cells highly expressing CXCR4 are generally found in organs such as liver, lung or bone. This suggests a privileged homing, survival and proliferation of metastatic breast cancer cells to these specific sites, where the local secretion of CXCL12 is strong [12], [18], [19]. Knockdown of CXCR4 by siRNA or blockage of CXCL12 binding with a CXCR4 specific neutralizing antibody or specific CXCR4 inhibitors impairs the proliferation and migration potential of these metastatic cells [19], [20].

Until recently, the ligand/receptor couple CXCL12/CXCR4 was thought to be exclusive because *cxcl12 −/−* and *cxcr4 −/−* mice had similar prenatal lethal phenotypes [21]. CXCL12, however, can also bind CXCR7, an orphan GPCR also called RDC1 [22], [23]. Growing evidence indicates a role for CXCR7 in cancer cell proliferation and migration [24], [25], [26], [27]. The relative expression levels of CXCR4 and CXCR7 may be critical to determine how the cell will respond to CXCL12. In fact, recent studies have shown that heterodimerization of these two receptors modulates the cellular response to CXCL12 [25], [28], [29].

In this study, we examined the regulation of the CXCL12/CXCR4/CXCR7 axis and its involvement in the proliferation and survival of E2-dependent and -independent breast cancer cells. Our results showed that the E2-dependent up-regulation of CXCL12 and CXCR4 that is associated with a down-regulation of CXCR7 could be pivotal for E2-induced growth of breast cancer cells.

## Materials and Methods

### 1. Antibodies and reagents

The antibodies used for the Western blot assays were rabbit polyclonal (Rp) antibody against CXCL12 (sc-28876; Santa Cruz Biotechnology, Heidelberg, Germany), Rp antibody against CXCR4 (ab2074; Abcam Inc., Cambridge, UK), Rp antibody against CXCR7 (ab12870; Abcam Inc.) and Rp antibody against ERK1 (sc-94; Santa Cruz Biotechnology). The antibodies used for FACS were murine monoclonal (Mm) antibody anti-human CXCR4 (clone 12G5, R&D Systems) and Mm antibody anti-human CXCR7/RDC1 (clone 11G8, R&D Systems, Minneapolis, MN, USA).

All reagents used for treatments were purchased from Sigma-Aldrich (St Louis, MO, USA): 17-β-estradiol (E2), ICI_182,.780_ (ICI), ethynyl-estradiol (EE2), Genistein (Gen), Chalcon 4 (inhibitor for CXCL12) and AMD3100 (inhibitor for CXCR4). CCX771 (inhibitor for CXCR7) was a kind gift from Dr. Mark Penfold (ChemoCentryx Inc., Mountain View, CA, USA).

### 2. Cell culture and treatments

The MCF-7 and ZR-75 (ER+) and the MDA-MB231 (ER−) human breast cancer cell lines were purchased from the American Type Culture Collection (Manassas, VA, USA). MCF-7, ZR-75 and MDA-MB-231 cells were routinely maintained in DMEM (Invitrogen, Cergy potoise, France) supplemented with 10% fetal bovine serum (FBS; Biowest, Paris, France) and antibiotics (Invitrogen) at 37°C in 5% CO_2_. When steroid treatments were required, the cells were maintained for 24 h in DMEM without phenol red (Invitrogen) supplemented with 2.5% dextran-treated charcoal stripped FBS (dsFBS) prior to the experiments. The treatments were then performed in DMEM phenol red-free 2.5% dsFBS during several periods of time with 0.1% ethanol as a control (EtOH), E2, ICI, EE2, or Gen.

### 3. RT-PCR assays

A total of 2.5×10^5^ MCF-7 cells was cultured in 6-well plates and treated as specified for each experiment. Total RNA from 3 independents wells per condition was extracted using Trizol™ (Invitrogen) according to the manufacturer's instructions. cDNAs were generated using MMLV Reverse transcriptase (Invitrogen) and random hexamers (Promega, Madison, WI, USA). Quantitative real-time RT-PCR was performed using the iQ SybrGreen supermix (BioRad, Hercules, CA, USA) on a BioRad MyiQ apparatus. The primers (Proligo Primers and Probes, Boulder, CO, USA) used for cDNA amplification in the quantitative RT-PCR experiments are described in Table S1.

### 4. Protein extraction/Western Blot

Cultures were performed in 10 cm diameter plates (80–90% confluence) and were treated for 48 h to 96 h, as specified for each experiment. Total proteins were extracted in RIPA buffer (1% NP40; 0.5% NaDeoxycholate; 1% SDS; in PBS) containing an anti-protease mix (Complete EDTA-free Antiproteases, Roche, Meylan, France), and protein concentration was measured using the Bio Rad DC protein assay kit. The proteins were diluted in Laemmli buffer and were denatured at 95°C. A total of 30 µg of the denatured proteins was then separated on SDS polyacrylamide gels (10 and 15%), transferred to a polyvinylidene difluoride membrane (Amersham, Uppsala, Sweden) and probed with specific antibodies. The immunocomplexes were detected using an enhanced chemiluminescence system (immune Star, Bio-Rad Laboratories).

### 5. ELISA

In all, 7×10^4^ MCF-7 cells were cultured in 24-well plates in 300 µL of medium. Cell culture supernatants from 6 independent well per condition were collected after 72 h of treatment. CXCL12 concentration was determined using the Quantikine kit (R&D Systems) according to the manufacturer's instructions.

### 6. Flow cytometry analysis

In all, 2.5×10^5^ MCF-7 cells were cultured in 6-well plates and treated for 48 h with EtOH or 10^−8^ M E2. For surface CXCR4 and CXCR7 detection, 5×10^5^ cells were incubated at 4°C for 45 min with 5 µg/ml of nonspecific isotype-matched controls, mouse to human IgG1 or mouse to human Ig2b or with 5 µg/ml of the specific monoclonal antibody to either CXCR4 or CXCR7. The cells were washed twice with PBS and were incubated with anti-Mouse PE (R-Phycoertythrin Goat anti-mouse) at 4°C for 45 min for nonspecific and CXCR7 binding. The cells were washed twice with PBS and were resuspended in 500 µL of PBS. In all, 10^4^ of cells from each sample were evaluated for fluorescence using the Cytomics FC500 apparatus (Beckman Coulter, Paris, France).

### 7. Formaldehyde-Assisted Isolation of Regulatory Elements (FAIRE)

FAIRE was performed as described by Eeckhoute *et al.* [30]. Briefly, asynchronously growing MCF-7 cells (60–70% confluence) treated or not for 48 h with 10^−8^ M E2 were cross-linked with 1% formaldehyde for 10 min at room temperature. Glycine was added to a final concentration of 125 mM, and the cells were rinsed with cold PBS and harvested. The cells were lysed with a solution of 1% SDS, 10 mM EDTA and 50 mM Tris-HCl (pH 8.1) containing a protease inhibitor cocktail (Roche) and were then sonicated for 14 min (30-sec on/off cycles) using a Bioruptor (Diagenode, Liège, Belgium) set at the highest intensity. The soluble chromatin was subjected to three consecutive phenol-chloroform extractions (Sigma, P3803) and incubated overnight at 65°C to reverse the cross-linking. The DNA was then purified using the MinElute PCR purification kit (Qiagen, Courtaboeuf, France). The relative enrichment of open chromatin for *CXCL12*, *CXCR4* and *CXCR7* proximal promoters was quantified by real-time PCR performed using the iQ SybrGreen supermix on a BioRad MyiQ apparatus. The primers used for the quantitative PCR experiments are described in Table S1.

### 8. Proliferation assays

All of the experiments involving the transient knockdown of CXCL12, CXCR4 or CXCR7 expression, the inhibition of CXCL12, CXCR4 or CXCR7 proteins using inhibitors and the transient over-expression of CXCR7 were carried out in MCF-7 cells.

In all, 2500 MCF-7 cells per well were seeded in 96-well plates one day before the siRNA transfection. The cells were then transfected in triplicate with siRNA targeting human CXCR4 and CXCR7 (Invitrogen) or human CXCL12 (Qiagen) using Lipofectamine 2000 (Invitrogen) according to the manufacturer's instructions. Cells transfected with nonspecific siRNA (Invitrogen) were used as controls. The day after transfection, the MCF-7 cells were cultured in 100 µL of medium with EtOH or 10^−8^ M E2 for 7 days. Every 2 days, the medium was removed, and fresh treatments were performed. When inhibitors were used, 2500 MCF-7 cells per well were seeded in 96-well plates. The treatments were then performed in 100 µL of medium with EtOH or 10^−8^ M E2 in combination with DMSO, Chalcon 4 (500 nM), AMD3100 (20 µM) or CCX771 (200 nM) for 7 days. As for the siRNA experiments, the medium was removed and fresh treatments were performed every 2 days. Relative cell number was evaluated using a 3-[4,5-dimethylthiazol-2-yl]-2,5-diphenyltetrazolium bromide (MTT; Sigma) assay. In all, 10 µL of the 5 mg/mL MTT solution was added to the 100 µL of culture medium in each well, and the cells were incubated for 2 h at 37°C. The supernatant was removed and the formazan formed was dissolved in 100 µL of DMSO. The absorbance of each well at 570 nm was obtained using a microplate reader. For the CXCR7 over-expression assays, 8000 MCF-7 cells were seeded in 24-well plates one day before transfection. The cells were then transiently transfected with 1 µg of pORF9-hCXCR7, an expression vector containing the human *CXCR7* open reading frame (InvivoGen, San Diego, CA, USA). The day after transfection, the cells were cultured in 500 µL of medium with EtOH or 10^−8^ M E2 for 7 days. Total cell number was evaluated by cell count using a Z2 COULTER COUNTER from Beckman Coulter. Each experiment was performed at least three times.

### 9. Statistical analysis

Statistical analysis was performed using the Student t-test. The values are provided as the mean ± the standard error of the mean (SEM) and were considered statistically significant for p<0.05.

## Results

### 1. The entire CXCL12 axis is an E2 endogenous target in ER-positive breast cancer cells

**Figure 1:**
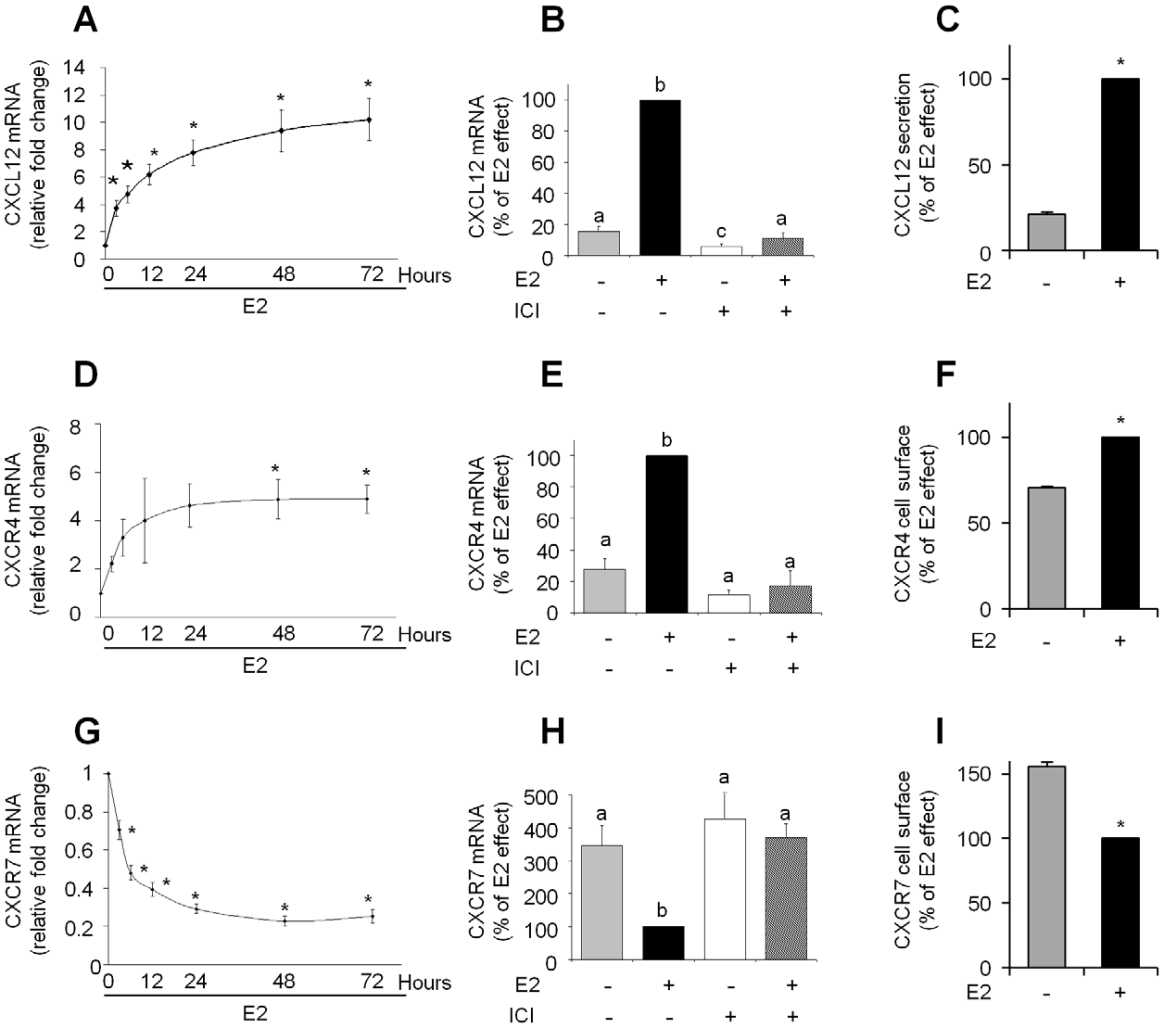
Regulation of CXCL12 signalization expression by E2 in MCF-7 cells. Cells were cultured as described in “Materials and Methods” and treated with E2 or ICI_184,780_ (ICI) using EtOH as vehicle. The vehicle-only treatment served as a control. The mRNA levels of *CXCL12* (A and B), *CXCR4* (D and E) and *CXCR7* (G and H) were quantified by real-time PCR analysis of cells treated for different periods of time with 10^−8^ M E2 (A, D and G) or cells treated for 48 h with 10^−8^ M E2 alone, 10^−6^ M ICI alone or both E2 and ICI (B, E and H). The real-time PCR results were normalized against the internal control *GAPDH* and expressed as the mean *CXCL12*, *CXCR4* or *CXCR7/GAPDH* mRNA ratio ± SEM of at least three independent experiments. Protein levels of CXCL12 (C), CXCR4 (F) and CXCR7 (I) was assayed. Secreted CXCL12 protein levels after treatment with EtOH or 10^−8^ M E2 for 48 h were determined by ELISA, and the values were normalized relative to the total protein concentration (C). The expression of CXCR4 and CXCR7 at the surface of MCF-7 cells was measured by flow cytometry after treatment with EtOH or 10^−8^ M E2 for 48 h (F, I). Representative data from at least three experiments performed in duplicate are shown. Asterisks or different lowercase letters indicate significant differences (*p*<0.05) between the control and treated cells.

The estrogenic regulation of CXCL12 was first confirmed in MCF-7 cells, a widely used model of ER-positive breast cancer cells. The cells were stimulated with 10^−8^ M E2 for different periods of time, and *CXCL12* mRNA was monitored using real-time quantitative RT-PCR. The positive regulation of CXCL12 in response to E2-treatment, which occurred in a time-dependent manner, was confirmed (Fig. 1A). The level of *CXCL12* transcripts significantly increased within 3 h (∼3-fold) and reached a maximum at 48 h (Fig. 1A). Both the basal and E2-induced expression levels of *CXCL12* decreased when the MCF-7 cells were co-treated with the pure anti-estrogen ICI_182,780_ (ICI) (Fig. 1B). Taken together, these findings indicate that ER is involved in basal and E2-induced *CXCL12* gene expression in MCF-7 cells. This E2 induction of CXCL12 was also confirmed at the protein level using ELISA assay (Fig. 1C). The addition of 10^−8^ M E2 significantly increased the secreted levels of the CXCL12 protein after 48 h of treatment when compared with the control cells (Fig. 1C).

The E2-regulation of CXCL12 receptors in breast cancer cells is largely unknown. Thus, CXCR4 and CXCR7 regulation by E2 was verified in our MCF-7 cells. *CXCR4* and *CXCR7* mRNA expression were monitored by real-time quantitative RT-PCR and both CXCR4 and CXCR7 receptors proteins were measured by FACS assays. Our results demonstrate that E2 stimulates the expression of *CXCR4* mRNA in MCF-7 cells (Fig. 1D). E2-stimulation of CXCR4 expression was significantly different to the control cells only after 48 h of E2-treatment. The 3 times induction was blocked by co-treatment with ICI, confirming that ER is involved in this regulation (Fig. 1E). In contrast, no effect was observed when MCF-7 cells were treated with ICI alone (Fig. 1E). Although earlier studies have shown that CXCR4 is weakly expressed at the surface of MCF-7 cells [31], our FACS assays revealed a reproducible and significant increase of CXCR4 protein by 40% after E2 treatment (Fig. 1F). Surprisingly, E2 was found to trigger a reduction of *CXCR7* mRNA levels in MCF-7 cells in a time-dependent manner (Fig. 1G). A significant down-regulation was observed only 3 h after E2-tretment, whereas the effect of E2 was reached the maximum after 48 h of treatment. This E2 effect was abolished when MCF-7 cells were co-treated with the pure anti-estrogen ICI, suggesting that ER is involved in this down-regulation (Fig. 1H). No effect was observed when MCF-7 cells were treated with ICI alone. The FACS analysis established that CXCR7 protein expression on the cell surface of E2-treated MCF-7 cells was significantly reduced by 40 to 45% compared with that in the solvent-treated control cells (Fig. 1I).

**Figure 2:**
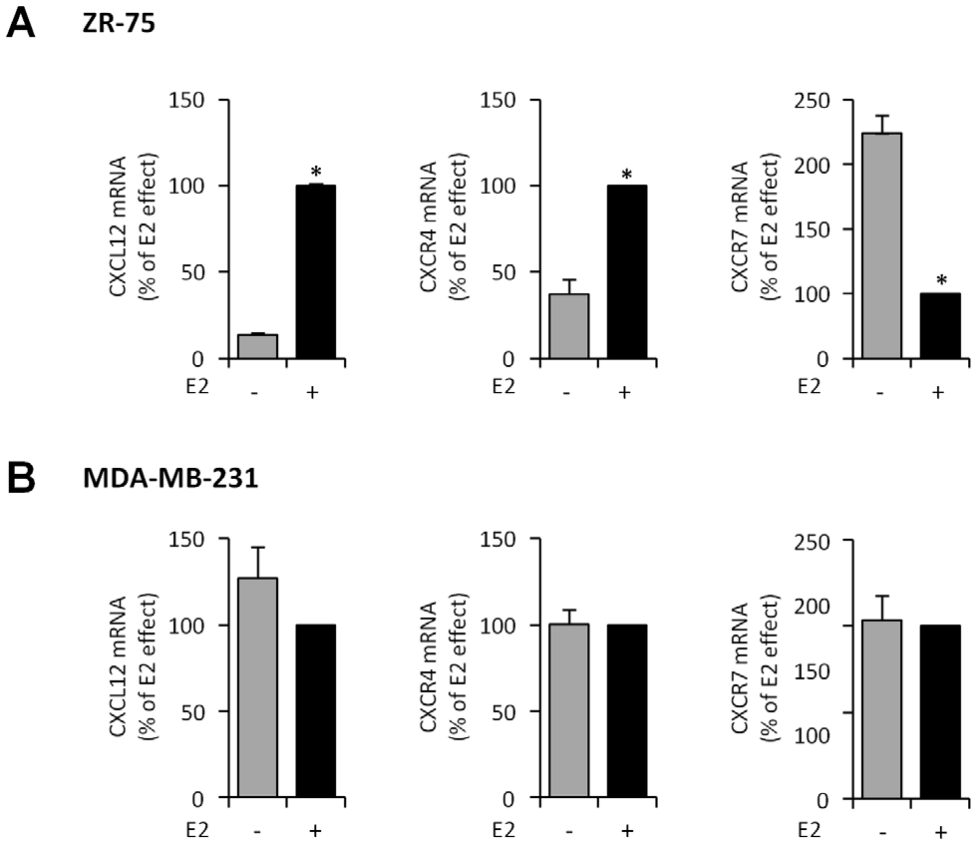
Regulation of *CXCL12*, *CXCR4* and *CXCR7* mRNA expressions by E2 in ZR-75 and MDA-MB-231 cells. *CXCL12*, *CXCR4* and *CXCR7* mRNA were assessed by quantitative real time PCR after 48 h treatment of ZR-75 (A) or MDA-MB-231 (B) cells to EtOH (−) or to10^−8^ M E2 (+). Transcript levels were normalized against *GAPDH* mRNA and data were calculated as percentage of the E2 effect. Data are from triplicate samples and are representative of three separate experiments. Asterisk indicates significant differences (*p*<0.05) between the control and ligand treated cells.

To investigate whether the differential E2-regulation of CXCR4 and CXCR7 could be extended to other breast cancer cell lines, we analyzed the expression of the components of the CXCL12 axis in ER-positive ZR-75 (Fig. 2A) and ER-negative MDA-MB-231 (Fig. 2B) breast cancer cell lines. As expected, we found a similar profile of regulation by E2 for *CXCL12*, *CXCR4* (which are induced) and *CXCR7* (which is repressed) mRNA in ZR-75 whereas these genes were not sensitive to E2 in MDA-MB-231 cells.

### 2. Effect of xeno-estrogens on the CXCL12/CXCR4/CXCR7 axis regulation in MCF-7 cells

**Figure 3:**
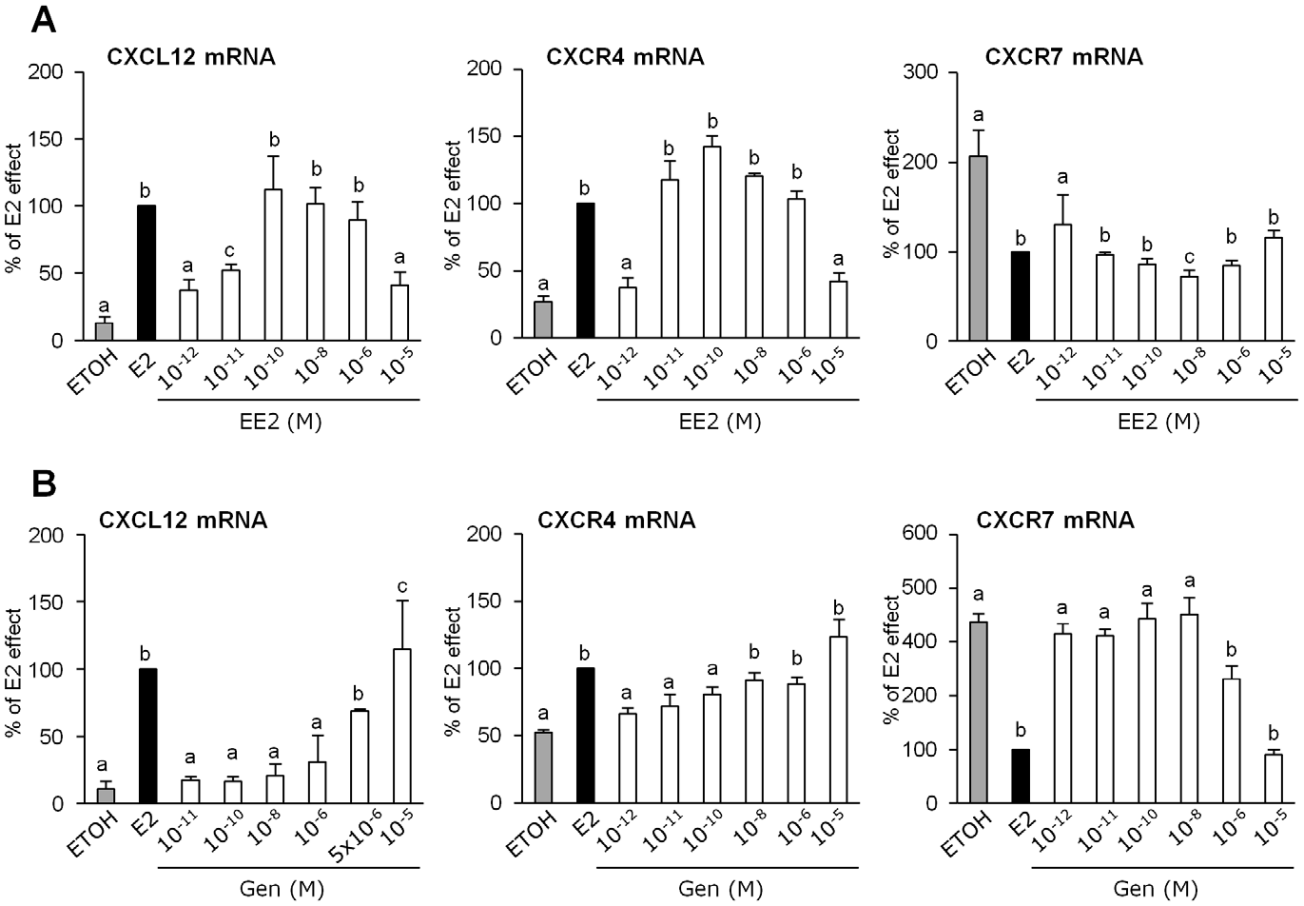
Xeno-estrogen effects on the expression of CXCL12, CXCR4 and CXCR7 in MCF-7 cells. The levels of the CXCL12, CXCR4 and CXCR7 transcripts were assessed by quantitative real-time PCR in MCF-7 cells treated under various conditions for 48 h. Treatment with EtOH and 10^−8^ M E2 served as the negative and positive controls, respectively. In each experimental assay, the cells were exposed to different concentrations of 17 α-ethynyl-estradiol (EE2) (A) and Genistein (Gen) (B). Transcript levels were normalized against *GAPDH* mRNA, and data were calculated as percentage of the E2 effect for each experiment. Significant differences (P<0.05) are indicated by different lowercase letters.

E2-target genes can be regulated differently by exogenous ER-ligand depending on the cell and promoter contexts. To examine whether the CXCL12 signaling axis may be differentially regulated by ER ligands in breast cancer cells, we examined the effects of several xeno-estrogens on the expression of CXCL12, CXCR4 and CXCR7 in MCF-7 cells. Using real-time RT-PCR, we assessed the effect of agonistic xeno-estrogens such as ethinyl-estradiol (EE2) and genistein (Gen) which are used in hormonal therapy. Dose-effect experiments were carried out and compared with 10^−8^ M of E2, the natural ligand (Fig. 3).

As expected, EE2 behaved as E2 as it induced significantly the expression of *CXCL12* and *CXCR4* mRNAs, whereas it decreased *CXCR7* mRNA expression (Fig. 3A). However, EE2 was much more potent since the maximal response obtained with EE2 required a concentration 10 to 100-fold lower than that required for E2 maximal effect. The phytoestrogen genistein also showed estrogenic properties on the CXCL12 axis (Fig. 3B). Nevertheless, high concentrations (from 10^−6^ M and 5×10^−6^ M) of ligand were required to significantly up regulate *CXCL12* or down regulate *CXCR7* expression. On the other hand, lower concentrations (from 10^−8^ M) of genistein were sufficient to stimulate *CXCR4* gene expression.

### 3. E2 modulates the chromatin structure of the *CXCL12*, *CXCR4* and *CXCR7* promoters

**Figure 4:**
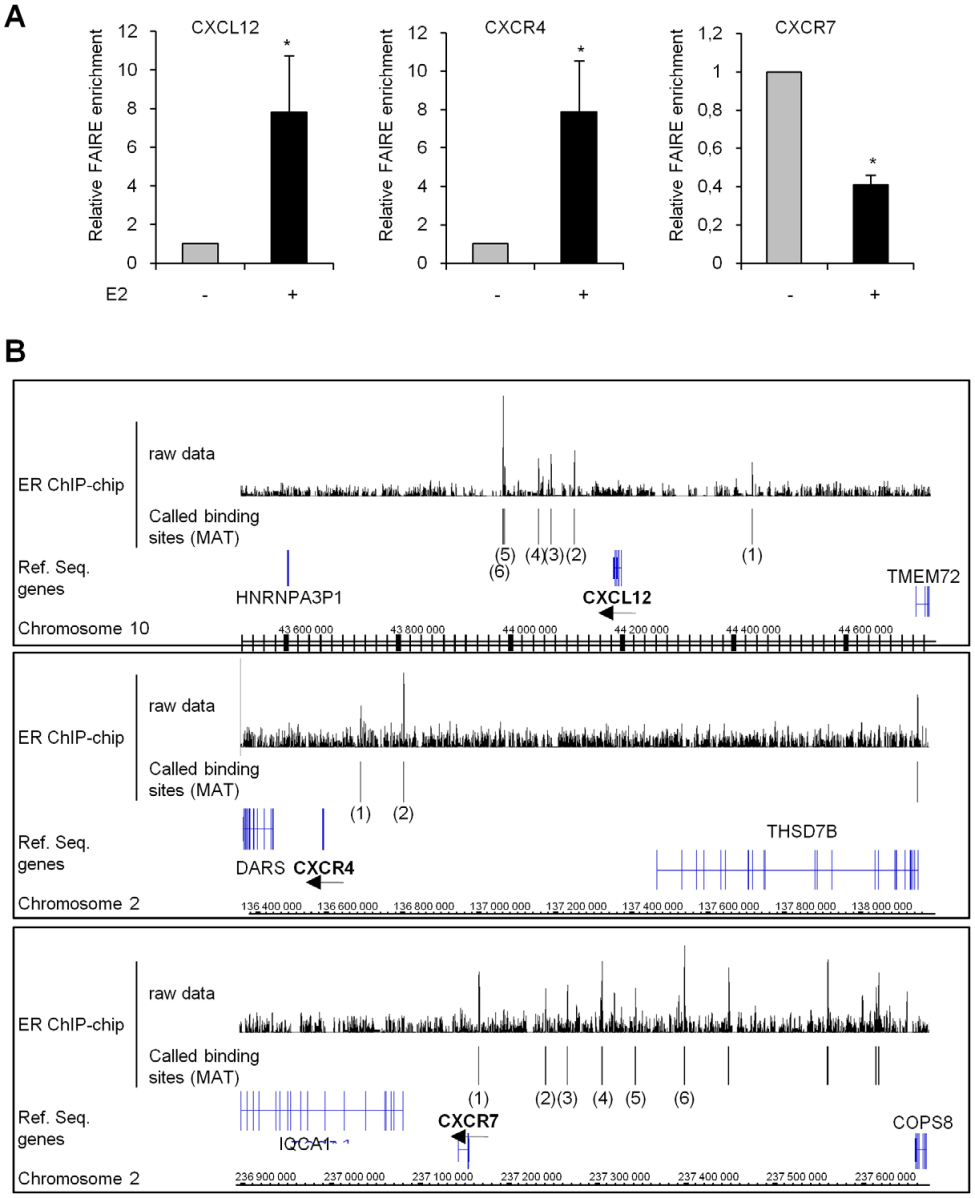
Impact of E2 treatment on the chromatin structure of the *CXCL12*, *CXCR4* and *CXCR7* promoters. (A) FAIRE assays were performed on MCF-7 cells exposed to either ETOH (−) or 10^−8^ M E2 (+) for 48 h. Real-time PCR was performed to monitor enrichment of the DNA corresponding to the proximal promoters of the *CXCL12*, *CXCR4* and *CXCR7* genes relative to input chromatin. The data are from triplicate samples and are representative of three separate experiments. Asterisks indicate significant differences (*p*<0.05) between the control and treated cells. (B) The Integrated Genome Browser (Affymetrix) was used to visualize ER-binding sites in the regions surrounding the *CXCL12*, *CXCR4* and *CXCR7* genes. Raw ChIP-chip data for ER and high confidence ER-binding sites called using the MAT algorithm are shown [34], [55]. The numbered ER-binding sites correspond to bound regions in which the closest TSS is that of *CXCL12*, *CXCR4* or *CXCR7*. Arrows indicate the orientation of the *CXCL12*, *CXCR4* and *CXCR7* genes.

The level of chromatin compaction appears to be well correlated with its activity. Recent studies have reported that active transcriptional regulatory sites are present within open chromatin regions in which the nucleosomes have been depleted [30]. These nucleosome-depleted genomic regions can be enriched from chromatin preparations using the FAIRE method [32]. Thus, FAIRE was used to monitor the effect of E2 on the chromatin structure of the *CXCL12*, *CXCR4* and *CXCR7* promoters in MCF-7 cells. E2 treatment (10^−8^ M) for 48 h resulted in an 8-fold increase in the amount of DNA corresponding to the *CXCL12* and *CXCR4* promoters in the FAIRE samples, indicating an opening of the chromatin at these two promoters due to E2 stimulation (Fig. 4A). In contrast, FAIRE enrichment of the *CXCR7* promoter was significantly decreased (∼60%) after E2 treatment of MCF-7 cells (Fig. 4A), suggesting that E2 triggers the chromatin containing the *CXCR7* promoter to be remodeled in a more condensed structure.

Zhu *et al.* showed recruitment of ERα to the *CXCL12* proximal promoter, which harbors an estrogen response element (ERE) half site [33]. Chromatin immunoprecipitation (ChIP) assays performed in this study, however, did not reveal any stable or reproducible recruitment of ERα to the *CXCL12*, *CXCR4* or *CXCR7* proximal promoters (up to 3 kb) (data not shown). Moreover, these proximal promoters of the *CXCL12*, *CXCR4* or *CXCR7* genes were not sensitive to E2 in luciferase-reporter assays performed in MCF-7 cells (data not shown). Recent studies on ER recruitment to the genome of breast cancer cells indicated that ER preferentially regulates its target genes by binding distal regulatory elements [34]. These distal regulatory sites can interact with the promoters of E2 target genes due to chromatin looping [35]. An examination of ChIP-chip data for ER from MCF-7 cells revealed that there were statistically significant sites surrounding the *CXCL12*, *CXCR4* and *CXCR7* genes where ER could bind (Fig. 4B). These three gene regions contain several ER-binding sites for which the closest transcription start sites (TSS) are those of *CXCL12*, *CXCR4* or *CXCR7*, which is a hallmark of E2-regulated genes [36]. The distance between the TSSs and the ER binding sites ranges from 20 to 250 kb, which is within the range of previously described active ER-bound enhancers [35], [37], [38], [39]. We have identified only one full ERE motif within the binding region, which is located 234 kb upstream from the TSS of the *CXCL12* gene (Fig S1), and several half ERE motifs in combination with SP1 and AP1 motifs within the genomic regions of the *CXCR4* and *CXCR7* genes (Fig S1).

### 4. Relationship between the CXCL12 axis and the basal and E2-dependent growth of MCF-7 cells

**Figure 5:**
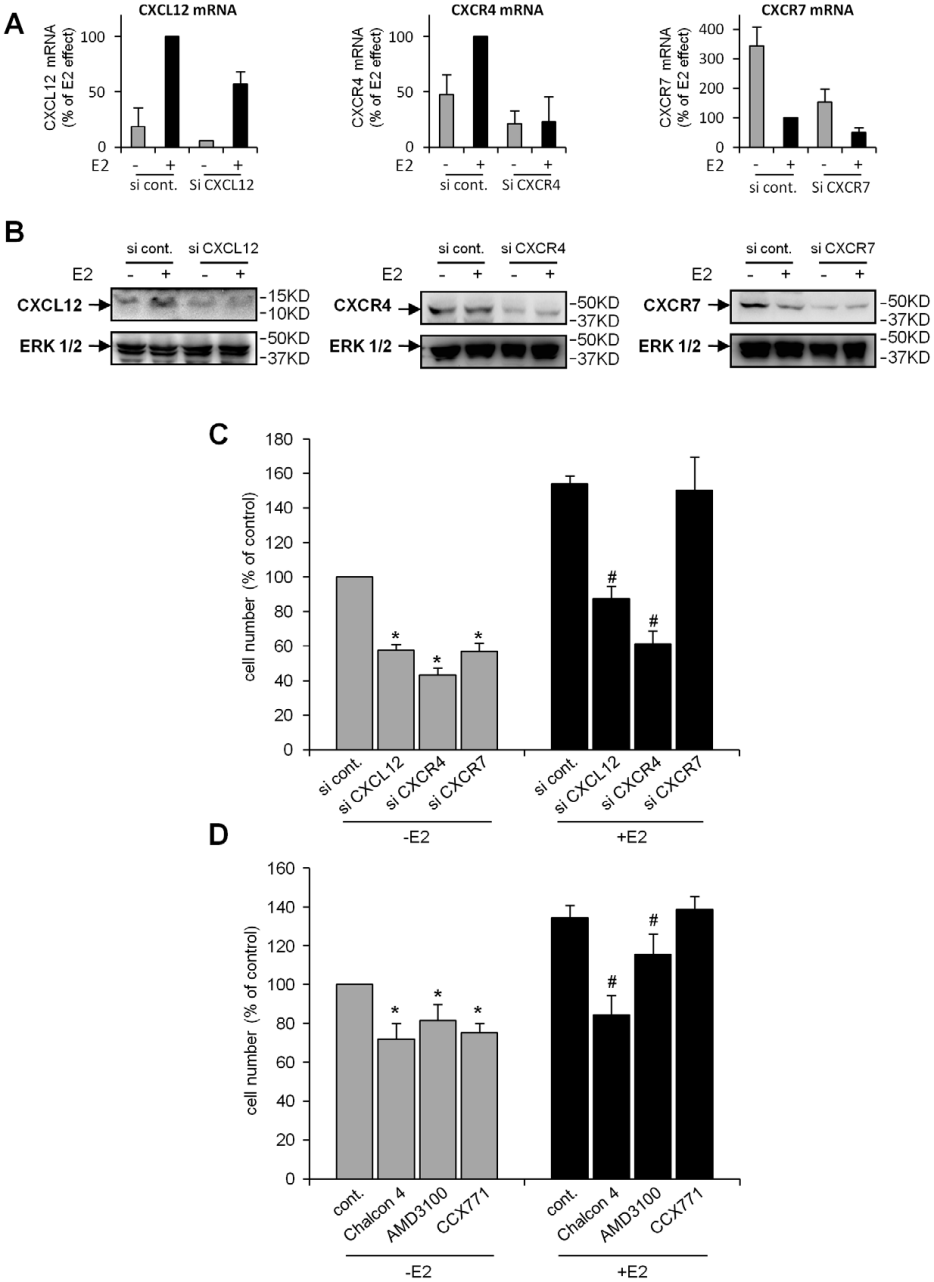
Involvement of CXCL12, CXCR4 and CXCR7 in the E2-dependent and -independent growth of MCF-7 cells. siRNA directed against CXCL12, CXCR4 or CXCR7 was transfected into MCF-7 cells treated with EtOH (−) or 10^−8^ M E2 (+). (A) After 48 h, the levels of the CXCL12, CXCR4 and CXCR7 transcripts were assessed by quantitative PCR and normalized against *GAPDH* mRNA. The results were compared with those obtained from MCF-7 cells transfected with a nonspecific siRNA control. (B) Total protein was extracted from MCF-7 cells, and the levels of CXCL12, CXCR4 and CXCR7 were analyzed by Western blotting. (C) To determine the growth rate of the MCF-7 cells, the siRNA-transfected cells were treated with EtOH (−E2) or 10^−8^ M E2 (+E2) for seven days. E2-dependent and -independent cell growth were evaluated using MTT assays of three independent experiments (n = 6). The results are expressed as a percentage of the relative cell number obtained from cells transfected with the control siRNA and treated with EtOH (considered as 100%). Significant differences (*p*<0.05) between transfected cells in the absence of E2 are indicated by an asterisk and between transfected cells in the presence of E2 by a sharp symbol. (D) The effects of specific inhibitors for CXCL12 (Chalcon 4), CXCR4 (AMD3100) or CXCR7 (CCX771) were measured after treatment of MCF-7 cells with either EtOH (−E2) or 10^−8^ M E2 (+E2) for 7 days. DMSO (vehicle) was used as the control. E2-dependent and E2-independent cell growth were then evaluated by MTT assays of three independent experiments (n = 6). The results are expressed as a percentage of the relative cell number obtained from cells treated with the vehicle control (considered as 100%). Significant differences (*p*<0.05) between treated cells in the absence of E2 are indicated by an asterisk and between treated cells in the presence of E2 by a sharp symbol.

CXCL12 is known to promote proliferation and survival of cancer cells in an autocrine and paracrine manner. Its regulation by E2 is also regarded as having an important role in the proliferative response to this hormone. However, the involvement of the entire CXCL12 signaling axis in cell growth is poorly documented. Specific siRNAs directed against CXCL12, CXCR4 and CXCR7 were used to assess the importance of this axis in the basal and E2-dependent growth of breast cancer cells. Quantitative RT-PCR and Western blot assays showed a knockdown efficiency of nearly 50%–60% compared with that of the control siRNA (Fig. 5A and 5B). MCF-7 cells transfected with the specific siRNAs were then exposed to 10^−8^ M E2 or solvent for 7 days, and the total cell number was quantified by MTT assay (Fig. 5C). The number of MCF-7 cells transfected with the specific siRNAs was reduced 45 to 60% compared with that of MCF-7 cells transfected with the siRNA control, suggesting that all three components of the CXCL12 axis are necessary for basal cell growth (Fig. 5C).

As expected, when the cells were transfected with the control siRNA, E2 treatment significantly increased the total cell number (1.63-fold) (Fig. 5C). When MCF-7 cells were transfected with siRNA directed against CXCL12, the total cell number in the presence of E2 reached only 45% of the control. Similarly, targeting CXCR4 decreased the total cell number in the presence of E2 (55%). In contrast, when decreasing CXCR7 expression by specific siRNA, the total cell number was comparable to E2-treated cells transfected with control siRNA (Fig. 5C).

To confirm these results, the effects of Chalcon 4, AMD3100 and CCX771, which are specific inhibitors of CXCL12, CXCR4 and CXCR7, respectively, were tested. The relative cell number was evaluated after 7 days of treatment with or without E2 in combination with DMSO or the different inhibitors. The results obtained with each specific inhibitor were generally similar to those obtained with each specific siRNA (Fig. 5C and 5D). The basal growth rate of MCF-7 cells was reduced after treatment with the Chalcon 4, AMD3100 or CCX771 inhibitors (Fig. 5D). In the presence of E2, Chalcon 4 and AMD3100 caused a significant reduction in cell growth, whereas CCX771 had no effect (Fig. 5D).

**Figure 6:**
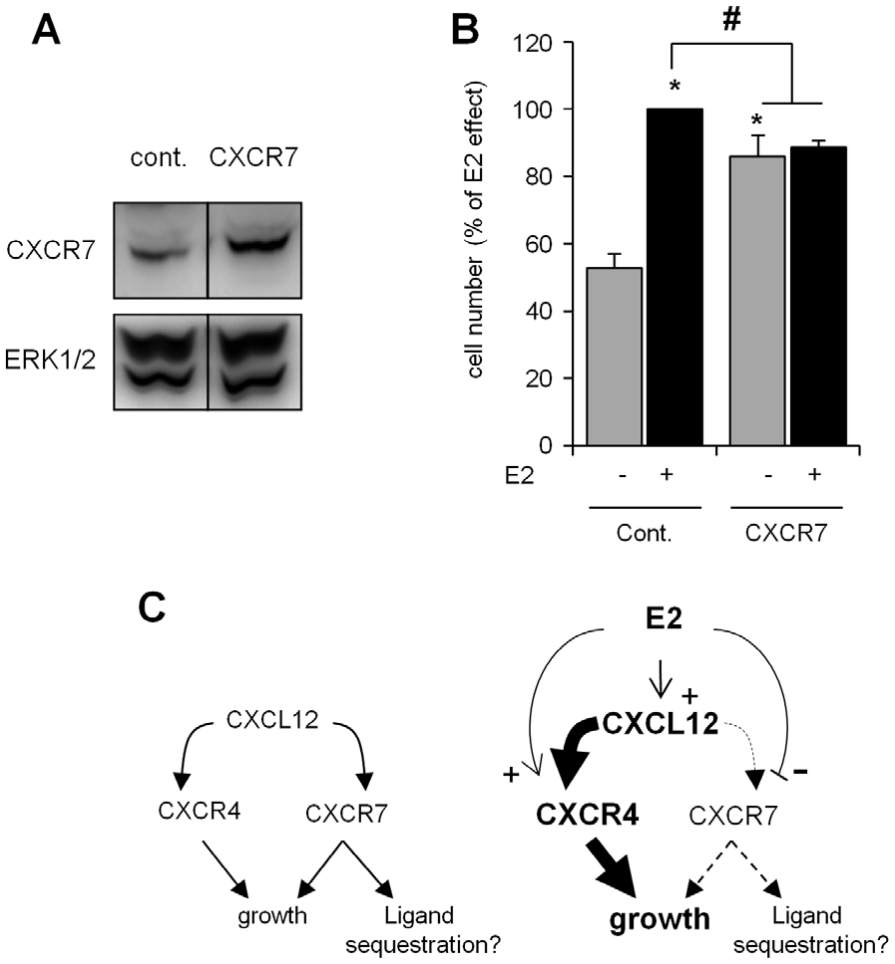
Impact of CXCR7 over-expression on the E2-dependent and -independent growth of MCF-7 cells. MCF-7 cells were transiently transfected with either a control expression vector or one containing the human *CXCR7* open reading frame. (A) Total protein extracts were prepared 48 h after transfection, and a Western blot analysis was performed to confirm CXCR7 over-expression. (B) Transfected cells were cultured in the presence of EtOH (−) or 10^−8^ M E2 (+) for seven days. E2-dependent and E2-independent cell growth rates were then evaluated by cell count of three independent experiments (n = 3). The results are expressed as a percentage of the relative cell number obtained from control cells treated with E2 (considered as 100%). Significant differences (*p*<0.05) between transfected cells in the absence of E2 are indicated by an asterisk and between transfected cells in the presence of E2 by a sharp symbol. (C) A proposed model for the involvement of the CXCL12 signaling axis in E2-dependent and -independent cell growth is shown. The binding of CXCL12 to CXCR4 and CXCR7 leads to the stimulation of cell growth through diverse pathways [28]. CXCR7 can also modulate CXCL12 availability by removing the chemokine from the extracellular space (left panel). Estrogens could stimulate cell growth by favoring the activation of CXCL12 through CXCR4 and reducing the expression of CXCR7 (right panel).

Furthermore, the impact of CXCR7 over-expression on MCF-7 cell growth was tested. Transient transfection of an expression vector containing human CXCR7, which was confirmed to produce elevated levels of CXCR7 protein (Fig. 6A), was sufficient to increase basal cell growth (Fig. 6B). Interestingly, this increase in CXCR7 expression significantly reduced the E2-induced growth effect (Fig. 6B). Thus, these findings suggest that although CXCR7 is likely involved in basal cell growth, the increased expression of this receptor may be unfavorable to the growth effect of E2 on MCF-7 cells.

## Discussion

The chemokine CXCL12 is thought to mediate the growth effect of E2 in ER-positive ovary and breast cancer cells [8]. CXCL12 binds to two G protein-coupled receptors, CXCR4 and CXCR7, which can modulate the response to CXCL12 by forming both homodimers and heterodimers. The regulation of CXCR4 and CXCR7 by estrogens and their involvement in the E2-dependent growth of breast cancer cells, however, have not been well characterized.

Our study showed that all of the components of the CXCL12 axis are targets of E2 in ER-positive but not in ER-negative breast cancer cells. Interestingly, our results showed that E2 differentially regulates both CXCL12 receptors. While CXCR4 expression was up-regulated by E2, CXCR7 expression was down-regulated by E2. A previous study conducted in Ishikawa endometrial adenocarcinoma cells have suggested that E2 could induce CXCR4 expression at the transcriptional level [15]. Regarding breast cancer cells, CXCR4 was also proposed to be induced by E2 but only through post-translational effects in a particular model of MCF-7 cells overexpressing HER2 [40]. Thus, we are the first to demonstrate that CXCR4 transcription can be induced by E2 stimulation in breast cancer cells. Moreover, to the best of our knowledge, the E2-induced down-regulation of CXCR7 has not been previously reported. The anti-estrogen ICI completely suppressed these effects by E2, indicating the importance of the classical nuclear ERs in the regulation of the entire CXCL12 signaling axis. It should be noticed that the discrepancy in the fold change factors observed between the chemokine receptors mRNA and protein levels, may argue for the consequence of additional control mechanisms besides transcription. This may be attributed to differences in the mRNA and protein turn over or could originate from different translational regulation. Moreover, following E2-treatment, the increased secretion of CXCL12 may modulate the internalization of both CXCR4 and CXCR7; and consequently may influence the expression of the chemokine receptors at the cell surface [22].

As expected, some xeno-estrogens such as EE2 (used in contraceptive pills) or genistein (a phytoestrogen found in food supplements), acted like E2 on the entire CXCL12 axis, with however different dose-effect profiles. These different dose-effects, notably regarding the discrepancies of genistein effect, might be linked to the importance of ER subtypes involved in the transcriptional regulations observed. Indeed, genistein is more ERβ than ERα selective and it is worth to note that there is very low level of ERβ expression in MCF-7 cells.

The FAIRE experiments confirmed the differential regulation of CXCR4 and CXCR7 by E2 and showed that this hormonal treatment affects the condensation state of the chromatin containing the proximal promoters of the *CXCL12*, *CXCR4* and *CXCR7* genes. Modification of the chromatin structure has been shown to correlate with the transcriptional potential of regulatory elements, and could suggest epigenetic modifications induced by E2 treatment.

The direct interaction of ER with the *CXCL12*, *CXCR4* and *CXCR7* genes was assessed using traditional ChIP and luciferase-reporter assays of the proximal promoter regions (up to 3 kb) in our MCF-7 cells. These analyses failed to show any ER binding sites or direct ER action at the proximal promoters of the *CXCL12*, *CXCR4* and *CXCR7* genes (data not shown). Nevertheless, the ChIP-chip analysis showed significant ER binding sites located 20–250 kb distal to the TSS of the *CXCL12*, *CXCR4* and *CXCR7* genes. These binding sites are always found closer to the TSS of the target gene than to the TSS of any other contiguous genes in the region. These results are in good agreement with recently published genome-wide studies showing that more than 90% of the mapped ER-binding sites are located far from the TSS of target genes and within intronic or distal regions (>5 kb from the 5′ and 3′ ends of adjacent transcripts). Moreover, the vast majority of these binding site sequences harbor full EREs, ERE-like or half ERE motifs [34], [41], [42], which were also found for the ER-binding sites associated with the *CXCL12*, *CXCR4* and *CXCR7* genes. Altogether, these finding suggest that direct interaction of ER with the *CXCL12*, *CXCR4* and *CXCR7* genes could occur primarily at these multiple distal sites, which would then allow ER to modulate transcription initiation at the target gene promoters by chromatin looping. Chromatin interactions have recently been proposed to represent a major mechanism for regulating gene transcription in mammals [35]. Accordingly, ER has been reported to function by extensive chromatin looping to provide a collaboration between outlying ER binding sites and other regulatory elements within proximal promoters that could be important for cell- and promoter-specific transcriptional regulation of target genes [35], [38].

E2 induces the expression of CXCL12 and CXCR4 and represses the expression of CXCR7 both at the transcriptional and translational levels, suggesting a functional regulation. The positive regulation of CXCL12 and CXCR4, which can induce cell proliferation and survival, has been well correlated with the growth effect of E2 on breast cancer cells. In contrast, the impact of the negative regulation of CXCR7 by E2 remains unclear. Recent studies have suggested that CXCR7 also contributes to cell proliferation or survival [23], [24], [27], [43]. Thus, the down regulation of its expression by E2 signaling would not be compatible with the growth effect of that hormone. We therefore examined the effect of the partial down-regulation or the inhibition of each component of the CXCL12 axis on the proliferation and survival of MCF-7 cells in the presence and absence of E2. Our results showed that each component of the CXCL12 axis contributes to the basal growth rate of MCF-7 cells. Each component of the axis, however, appears to contribute differently to the E2-dependent growth of MCF-7 cells. In agreement with a previous study [8], we also observed that reducing CXCL12 expression or inhibiting its activity significantly limits the E2-induced growth effect. Similarly, inhibition by CXCR4-specific siRNA or by AMD3100, a specific inhibitor of this receptor, significantly decreased the stimulation of cell growth by E2. Taken together, these observations indicate that enhancement of the expression of CXCL12 and CXCR4 by E2 may constitute a molecular mechanism by which breast cancer cells proliferate in response to that hormone. Although in this study, we measured the global effect of E2 on cell growth which is a resultant of both cell survival and proliferation, future work by testing cell cycle distribution would be necessary to determine the specific cell cycle phase.

Several studies have demonstrated that CXCR7 does not function like a classical GPCR, and this receptor could mediate intracellular CXCL12 signaling via a different mechanism than that of the CXCL12-CXCR4 complex [28], [43], [44]. Although the reduction of CXCR7 expression or inhibition of its activity, which reproduce the natural effect of that hormone, did not modify the E2-dependent proliferation of MCF-7 cells, its over-expression in MCF-7 cells affected basal cell growth positively and E2-dependent induction of cell growth negatively. These observations suggest that the E2-induced down-regulation of CXCR7 could be associated with the effect of this hormone on breast cancer cell growth. Thus, the contribution of CXCR7 to cell proliferation would be different depending on whether cell proliferation was promoted by E2 or by other growth factors. Recently, Luker *et al.* showed that CXCR7 can modulate the availability of CXCL12 by its removal from the extracellular space [45]. This mechanism could limit CXCL12 signaling via CXCR4. Thus, a decrease in the cell surface expression of CXCR7 and an augmentation of CXCL12 secretion and CXCR4 expression by E2 could promote cell proliferation by favoring CXCL12 signaling through CXCR4 (Fig. 6C). Nevertheless, this particular role for the scavenger receptor, which has also been reported in other cancer cell and animal models, might not represent the only mode of action for CXCR7. Indeed, the CXCR4 and CXCR7 receptors appear to be constitutively able to form homo- and heterodimers that could modulate the sensitivity and response to the CXCL12 ligand [46], [47], [48]. Furthermore, the formation of homo- or heterodimers seems to depend primarily on the expression level of both receptors in the cells [25]. The differential regulation of both CXCL12 receptors by estrogens would therefore have important consequences. The expression level of CXCR7 could serve as an important element that facilitates CXCR4 in the signal transduction of CXCL12. By specifically modifying CXCR4 positively and CXCR7 negatively, we speculate that E2 could modulates the ratio of the two GPRCs, promoting the formation of CXCR4/CXCR7 heterodimers or CXCR4/CXCR4 homodimers on the surface of MCF-7 cells, which would create an environment favorable to the stimulation of cell growth.

Expression profiling studies showed that the highest ER expression levels was found in tumors associated with the most favorable survival outcomes [49], [50], [51]. The expression of ERs in breast cancer cells prevents the acquisition of a high potential for the migration and invasion of tumor cells by promoting the maintenance of these cells in a differentiated state [5], [6]. In addition to its involvement in cell proliferation, the signaling axis of CXCL12 is strongly associated with cell migration [13], [18], [20]. Recently, the autocrine/paracrine CXCL12 stimulation of cancer cells was reported to restrain their migration behavior. The loss of local CXCL12 expression may then be necessary to allow the cells to spread within the organism toward endocrine sources of CXCL12 [52], [53], [54]. During cancer progression, the hormonal control of the CXCL12 signaling axis in breast cancer cells may therefore play major roles in tumor growth and the suppression of the invasion potential of cancer cells.

In conclusion, our study demonstrated that the components of the entire CXCL12 signaling axis are targeted by E2 in breast cancer cells. The E2-induced up-regulation of the chemokine CXCL12 and its receptor CXCR4 and down-regulation of CXCR7 could be associated with the effect of estrogens on the growth of breast cancer cells.

## Supporting Information

Figure S1 **Sequence analysis and localizations of ER binding sites in the distal genomic regions of CXCL12, CXCR4 and CXCR7 genes.** Genomic sequence regions corresponding to which significant ER binding sites are identified by ChIP-chip for CXCL12 gene (on chromosome 12), CXCR4 and CXCR7 (on chromosome 2) are shown. As also indicated in Fig. 4 B, sequences of the six ER binding sites for CXCL12 gene located at 234 kb upstream and 83 kb, 125 kb, 147 kb, 208 kb and 210 kb downstream of TSS.; as well as the two sites found for CXCR4 gene located at 97 kb and 210 kb upstream from TSS; and the six sites found for CXCR7 gene located at 23 kb, 99 kb, 124 kb, 163 kb, 200 kb and 256 Kb upstream from TSS are indicated. Using the TESS web based software (Transcription Element Search System; www.cbil.upenn.edu/cgi-bin/tess), the putative binding sites for transcription factors were examined in these genomic regions. In addition to only one full ERE motif found within the binding region located at 234 kb upstream from the TSS of CXCL12 gene, principally half ERE, SP1 and AP1 motifs were found within these genomic regions.
(TIF)

Table S1 **Primers sequences.**
(TIF)

## References

[1] Deroo BJ, Korach KS (2006). Estrogen receptors and human disease.. J Clin Invest.

[2] Zhao C, Dahlman-Wright K, Gustafsson JA (2008). Estrogen receptor beta: an overview and update.. Nucl Recept Signal.

[3] Sommer S, Fuqua SA (2001). Estrogen receptor and breast cancer.. Semin Cancer Biol.

[4] Wickerham DL, Costantino JP, Vogel VG, Cronin WM, Cecchini RS (2009). The use of tamoxifen and raloxifene for the prevention of breast cancer.. Recent Results Cancer Res.

[5] Platet N, Cunat S, Chalbos D, Rochefort H, Garcia M (2000). Unliganded and liganded estrogen receptors protect against cancer invasion via different mechanisms.. Mol Endocrinol.

[6] Rochefort H, Platet N, Hayashido Y, Derocq D, Lucas A (1998). Estrogen receptor mediated inhibition of cancer cell invasion and motility: an overview.. J Steroid Biochem Mol Biol.

[7] Foster JS, Henley DC, Ahamed S, Wimalasena J (2001). Estrogens and cell-cycle regulation in breast cancer.. Trends Endocrinol Metab.

[8] Hall JM, Korach KS (2003). Stromal cell-derived factor 1, a novel target of estrogen receptor action, mediates the mitogenic effects of estradiol in ovarian and breast cancer cells.. Mol Endocrinol.

[9] Kishimoto H, Wang Z, Bhat-Nakshatri P, Chang D, Clarke R (2005). The p160 family coactivators regulate breast cancer cell proliferation and invasion through autocrine/paracrine activity of SDF-1alpha/CXCL12.. Carcinogenesis.

[10] Kucia M, Jankowski K, Reca R, Wysoczynski M, Bandura L (2004). CXCR4-SDF-1 signalling, locomotion, chemotaxis and adhesion.. J Mol Histol.

[11] Miller RJ, Banisadr G, Bhattacharyya BJ (2008). CXCR4 signaling in the regulation of stem cell migration and development.. J Neuroimmunol.

[12] Burger JA, Kipps TJ (2006). CXCR4: a key receptor in the crosstalk between tumor cells and their microenvironment.. Blood.

[13] Luker KE, Luker GD (2006). Functions of CXCL12 and CXCR4 in breast cancer.. Cancer Lett.

[14] Smith MC, Luker KE, Garbow JR, Prior JL, Jackson E (2004). CXCR4 regulates growth of both primary and metastatic breast cancer.. Cancer Res.

[15] Kubarek L, Jagodzinski PP (2007). Epigenetic up-regulation of CXCR4 and CXCL12 expression by 17 beta-estradiol and tamoxifen is associated with formation of DNA methyltransferase 3B4 splice variant in Ishikawa endometrial adenocarcinoma cells.. FEBS Lett.

[16] Andre F, Cabioglu N, Assi H, Sabourin JC, Delaloge S (2006). Expression of chemokine receptors predicts the site of metastatic relapse in patients with axillary node positive primary breast cancer.. Ann Oncol.

[17] Su YC, Wu MT, Huang CJ, Hou MF, Yang SF (2006). Expression of CXCR4 is associated with axillary lymph node status in patients with early breast cancer.. Breast.

[18] Kang H, Watkins G, Parr C, Douglas-Jones A, Mansel RE (2005). Stromal cell derived factor-1: its influence on invasiveness and migration of breast cancer cells in vitro, and its association with prognosis and survival in human breast cancer.. Breast Cancer Res.

[19] Muller A, Homey B, Soto H, Ge N, Catron D (2001). Involvement of chemokine receptors in breast cancer metastasis.. Nature.

[20] Liang Z, Wu H, Reddy S, Zhu A, Wang S (2007). Blockade of invasion and metastasis of breast cancer cells via targeting CXCR4 with an artificial microRNA.. Biochem Biophys Res Commun.

[21] Ma Q, Jones D, Borghesani PR, Segal RA, Nagasawa T (1998). Impaired B-lymphopoiesis, myelopoiesis, and derailed cerebellar neuron migration in CXCR4- and SDF-1-deficient mice.. Proc Natl Acad Sci U S A.

[22] Balabanian K, Lagane B, Infantino S, Chow KY, Harriague J (2005). The chemokine SDF-1/CXCL12 binds to and signals through the orphan receptor RDC1 in T lymphocytes.. J Biol Chem.

[23] Burns JM, Summers BC, Wang Y, Melikian A, Berahovich R (2006). A novel chemokine receptor for SDF-1 and I-TAC involved in cell survival, cell adhesion, and tumor development.. J Exp Med.

[24] Wang J, Shiozawa Y, Wang J, Wang Y, Jung Y (2008). The role of CXCR7/RDC1 as a chemokine receptor for CXCL12/SDF-1 in prostate cancer.. J Biol Chem.

[25] Levoye A, Balabanian K, Baleux F, Bachelerie F, Lagane B (2009). CXCR7 heterodimerizes with CXCR4 and regulates CXCL12-mediated G protein signaling.. Blood.

[26] Zabel BA, Wang Y, Lewen S, Berahovich RD, Penfold ME (2009). Elucidation of CXCR7-mediated signaling events and inhibition of CXCR4-mediated tumor cell transendothelial migration by CXCR7 ligands.. J Immunol.

[27] Yoshida D, Nomura R, Teramoto A (2009). Signaling Pathway Mediated by CXCR7, an Alternative Chemokine Receptor for Stromal-Cell Derived Factor-1alpha, in AtT20 mouse ACTH-secreting pituitary adenoma cells.. J Neuroendocrinol.

[28] Thelen M, Thelen S (2008). CXCR7, CXCR4 and CXCL12: an eccentric trio?. J Neuroimmunol.

[29] Springael JY, Urizar E, Parmentier M (2005). Dimerization of chemokine receptors and its functional consequences.. Cytokine Growth Factor Rev.

[30] Eeckhoute J, Lupien M, Meyer CA, Verzi MP, Shivdasani RA (2009). Cell-type selective chromatin remodeling defines the active subset of FOXA1-bound enhancers.. Genome Res.

[31] Dewan MZ, Ahmed S, Iwasaki Y, Ohba K, Toi M (2006). Stromal cell-derived factor-1 and CXCR4 receptor interaction in tumor growth and metastasis of breast cancer.. Biomed Pharmacother.

[32] Nagy PL, Cleary ML, Brown PO, Lieb JD (2003). Genomewide demarcation of RNA polymerase II transcription units revealed by physical fractionation of chromatin.. Proc Natl Acad Sci U S A.

[33] Zhu Y, Sullivan LL, Nair SS, Williams CC, Pandey AK (2006). Coregulation of estrogen receptor by ERBB4/HER4 establishes a growth-promoting autocrine signal in breast tumor cells.. Cancer Res.

[34] Carroll JS, Meyer CA, Song J, Li W, Geistlinger TR (2006). Genome-wide analysis of estrogen receptor binding sites.. Nat Genet.

[35] Fullwood MJ, Liu MH, Pan YF, Liu J, Xu H (2009). An oestrogen-receptor-alpha-bound human chromatin interactome.. Nature.

[36] Krum SA, Miranda-Carboni GA, Lupien M, Eeckhoute J, Carroll JS (2008). Unique ERalpha cistromes control cell type-specific gene regulation.. Mol Endocrinol.

[37] Carroll JS, Liu XS, Brodsky AS, Li W, Meyer CA (2005). Chromosome-wide mapping of estrogen receptor binding reveals long-range regulation requiring the forkhead protein FoxA1.. Cell.

[38] Eeckhoute J, Carroll JS, Geistlinger TR, Torres-Arzayus MI, Brown M (2006). A cell-type-specific transcriptional network required for estrogen regulation of cyclin D1 and cell cycle progression in breast cancer.. Genes Dev.

[39] Boney-Montoya J, Ziegler YS, Curtis CD, Montoya JA, Nardulli AM (2009). Long-range transcriptional control of progesterone receptor gene expression.. Mol Endocrinol.

[40] Sengupta S, Schiff R, Katzenellenbogen BS (2009). Post-transcriptional regulation of chemokine receptor CXCR4 by estrogen in HER2 overexpressing, estrogen receptor-positive breast cancer cells.. Breast Cancer Res Treat.

[41] Lin CY, Vega VB, Thomsen JS, Zhang T, Kong SL (2007). Whole-genome cartography of estrogen receptor alpha binding sites.. PLoS Genet.

[42] Charn TH, Liu ET, Chang EC, Lee YK, Katzenellenbogen JA (2010). Genome-wide dynamics of chromatin binding of estrogen receptors alpha and beta: mutual restriction and competitive site selection.. Mol Endocrinol.

[43] Mazzinghi B, Ronconi E, Lazzeri E, Sagrinati C, Ballerini L (2008). Essential but differential role for CXCR4 and CXCR7 in the therapeutic homing of human renal progenitor cells.. J Exp Med.

[44] Hartmann TN, Grabovsky V, Pasvolsky R, Shulman Z, Buss EC (2008). A crosstalk between intracellular CXCR7 and CXCR4 involved in rapid CXCL12-triggered integrin activation but not in chemokine-triggered motility of human T lymphocytes and CD34+ cells.. J Leukoc Biol.

[45] Luker KE, Steele JM, Mihalko LA, Ray P, Luker GD (2010). Constitutive and chemokine-dependent internalization and recycling of CXCR7 in breast cancer cells to degrade chemokine ligands.. Oncogene.

[46] Luker KE, Gupta M, Luker GD (2009). Imaging chemokine receptor dimerization with firefly luciferase complementation.. Faseb J.

[47] Sierro F, Biben C, Martinez-Munoz L, Mellado M, Ransohoff RM (2007). Disrupted cardiac development but normal hematopoiesis in mice deficient in the second CXCL12/SDF-1 receptor, CXCR7.. Proc Natl Acad Sci U S A.

[48] Sohy D, Parmentier M, Springael JY (2007). Allosteric transinhibition by specific antagonists in CCR2/CXCR4 heterodimers.. J Biol Chem.

[49] Bergamaschi A, Hjortland GO, Triulzi T, Sorlie T, Johnsen H (2009). Molecular profiling and characterization of luminal-like and basal-like in vivo breast cancer xenograft models.. Mol Oncol.

[50] Morrow PK, Hortobagyi GN (2009). Management of breast cancer in the genome era.. Annu Rev Med.

[51] Rennstam K, McMichael N, Berglund P, Honeth G, Hegardt C (2009). Numb protein expression correlates with a basal-like phenotype and cancer stem cell markers in primary breast cancer.. Breast Cancer Res Treat.

[52] Zhou W, Jiang Z, Liu N, Xu F, Wen P (2009). Down-regulation of CXCL12 mRNA expression by promoter hypermethylation and its association with metastatic progression in human breast carcinomas.. J Cancer Res Clin Oncol.

[53] Zhou W, Jiang Z, Song X, Liu Y, Wen P (2008). Promoter hypermethylation-mediated down-regulation of CXCL12 in human astrocytoma.. J Neurosci Res.

[54] Wendt MK, Cooper AN, Dwinell MB (2008). Epigenetic silencing of CXCL12 increases the metastatic potential of mammary carcinoma cells.. Oncogene.

[55] Lupien M, Eeckhoute J, Meyer CA, Wang Q, Zhang Y (2008). FoxA1 translates epigenetic signatures into enhancer-driven lineage-specific transcription.. Cell.

