# Supplementary figures and images for: Differential Estrogen-Regulation of CXCL12 Chemokine Receptors, CXCR4 and CXCR7, Contributes to the Growth Effect of Estrogens in Breast Cancer Cells
Source: PLoS One. 2011 Jun 10;6(6):e20898. doi: 10.1371/journal.pone.0020898 (PMC3112227; doi:10.1371/journal.pone.0020898)

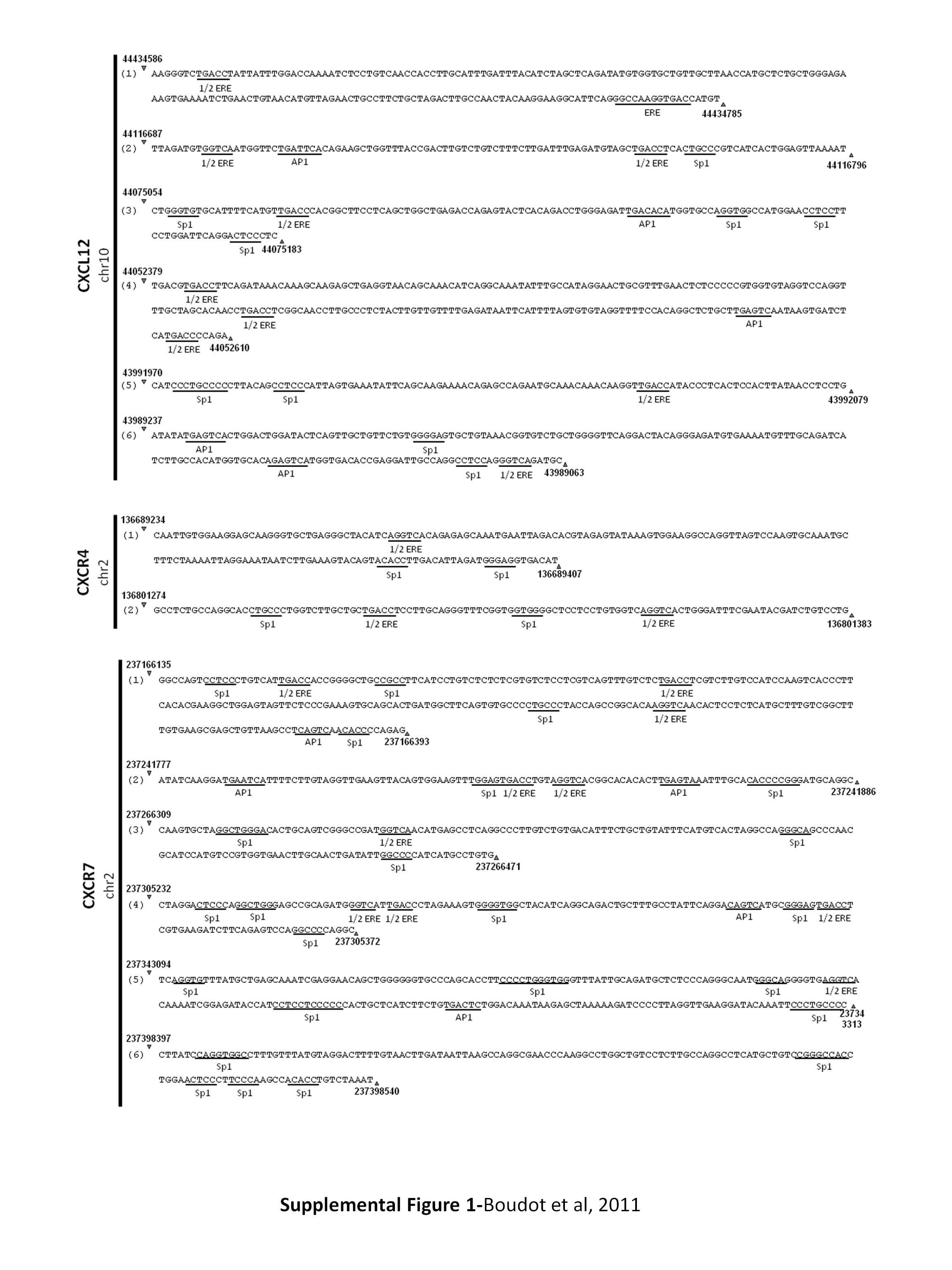

Supplement: Figure S1 — Sequence analysis and localizations of ER binding sites in the distal genomic regions of CXCL12, CXCR4 and CXCR7 genes. Genomic sequence regions corresponding to which significant ER binding sites are identified by ChIP-chip for CXCL12 gene (on chromosome 12), CXCR4 and CXCR7 (on chromosome 2) are shown. As also indicated in Fig. 4 B, sequences of the six ER binding sites for CXCL12 gene located at 234 kb upstream and 83 kb, 125 kb, 147 kb, 208 kb and 210 kb downstream of TSS.; as well as the two sites found for CXCR4 gene located at 97 kb and 210 kb upstream from TSS; and the six sites found for CXCR7 gene located at 23 kb, 99 kb, 124 kb, 163 kb, 200 kb and 256 Kb upstream from TSS are indicated. Using the TESS web based software (Transcription Element Search System; www.cbil.upenn.edu/cgi-bin/tess), the putative binding sites for transcription factors were examined in these genomic regions. In addition to only one full ERE motif found within the binding region located at 234 kb upstream from the TSS of CXCL12 gene, principally half ERE, SP1 and AP1 motifs were found within these genomic regions. (TIF) [file pone.0020898.s001.tif]

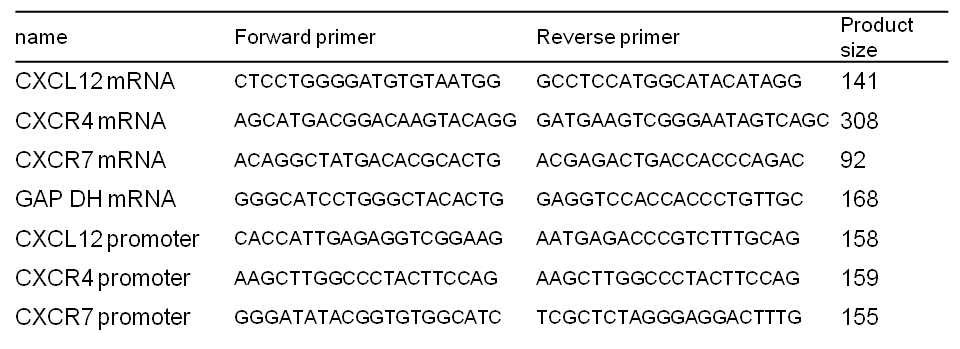

Supplement: Table S1 — Primers sequences. (TIF) [file pone.0020898.s002.tif]
